# Fewer neurocognitive deficits and less brain atrophy by third ventricle measurement in PLWH treated with modern ART: A prospective analysis

**DOI:** 10.3389/fneur.2022.962535

**Published:** 2022-08-23

**Authors:** Dominic Kaddu-Mulindwa, Matthias Heit, Gudrun Wagenpfeil, Moritz Bewarder, Klaus Fassbender, Stefanie Behnke, Umut Yilmaz, Mathias Fousse

**Affiliations:** ^1^Department of Hematology and Oncology, Saarland University Medical School, Homburg, Germany; ^2^Institute for Medical Biometrics, Epidemiology and Medical Computer Science, Saarland University Medical School, Homburg, Germany; ^3^Department of Neurology, Saarland University Medical School, Homburg, Germany; ^4^Department of Neuroradiology, Saarland University Medical School, Homburg, Germany

**Keywords:** HIV, third ventricle diameter, transcranial ultrasound, HIV associated neurocognitive disorders, fractional anisotropy, CERAD-PLUS

## Abstract

**Background:**

Despite antiretroviral therapy, cognitive dysfunction seems to remain a major issue for people living with human immunodeficiency virus (PLWH). Previous studies showed a correlation between the width of the third ventricle (WTV) and neurocognitive disorders in PLWH.

**Patients and methods:**

We investigated prevalence and correlation of neuropsychological disorders using WTV as a brain atrophy marker examined by transcranial sonography and MRI in PLWH and healthy age- and gender-matched controls. We used Becks Depression Inventory (BDI) for depression screening, the questionnaires Fatigue Severity Scale (FSS) for fatigue and Short-Form-36 (SF36) for quality of life (QoL) evaluation and Consortium to establish a registry for Alzheimer's disease (CERAD-PLUS) as neuropsychological test battery.

**Results:**

52 PLWH (47 males) and 28 non-infected controls (23 males) with a median age of 52 years (24–78 years) and 51 years (22–79) were analyzed. WTV correlated significantly with age (*p* < 0.01) but showed no significantly difference in PLWH (median = 3.4 mm) compared to healthy controls (median = 2.8 mm) (*p* = 0.085). PLWH had both significantly higher BDI-Scores (*p* = 0.005) and FSS-Scores (*p* = 0.012). Controls reported higher QoL (SF-36) with significant differences in most items. However, the overall cognitive performance (CERAD total score) showed no significant difference. The WTV of all subjects correlated with neurocognitive performance measured as CERAD total score (*p* = 0.009) and trail making tests A (*p* < 0.001) and B (*p* = 0.018). There was no correlation between the scores of BDI, FSS, SF-36, and CERAD-PLUS items and WTV.

**Conclusion:**

WTV is considered as a predictor of cognitive deficits in neurodegenerative diseases. Nevertheless, we found no significant difference in WTV or overall cognitive performance between PLWH and controls. PLWH suffer more often from depression and fatigue and report reduced QoL when compared to healthy controls.

## Introduction

Due to the introduction of antiretroviral therapy (ART) in 1996, the life expectancy for people living with human immunodeficiency virus (PLWH, HIV) increased over the last 30 years up to nearly normal compared to non-infected individuals nowadays (1, 2). In addition, because of ART, the incidence of opportunistic infections and HIV-associated deaths decreased (3). Nevertheless, the incidence of HIV-associated neurocognitive disorders (HAND), which includes the entire spectrum of cognitive disorders associated with HIV-1 infection was found to be less decreasing compared to other AIDS-defining conditions (4), although other neurologic disorders like severe forms of HIV-associated dementia are much rarer nowadays. Even with ART, the prevalence of HAND still appears to be 30–50% (5, 6), with a decrease in HIV-associated dementia (HAD) with a concomitant relative increase in milder stages when compared to the pre-ART-era (7). However, cognitive dysfunction still remains a relevant issue for PLWH and can affect their daily quality of life (QoL) (8). In daily clinical practice, it could be sometimes very difficult and time-consuming to set up the diagnosis of HAND, because of the broad spectrum of disorders, although diagnostic criteria for HAND exist to guide physicians in their daily routine (9).

Hence, it is important to detect even subtle changes of neurocognitive function or other indicators of brain alteration in PLWH, revealing the need to initiate ART in treatment naive patients or probably switch ART-regime. This is also in view of the fact that ART itself might contribute to cognitive dysfunction (10). Diagnosing especially milder forms, e.g., asymptomatic neurocognitive impairment (ANI) and mild neurocognitive disorder (MND), is complicated by the need of performing time-consuming test batteries in which an acquired deficit in at least two cognitive performances (verbal fluency, executive functions, information processing speed, attention, working memory, or verbal and visual learning) must be demonstrated in two standardized tests. In addition, questions about the social environment regarding everyday life limitations in various areas (inefficiency in work, homemaking, social functioning or mental acuity) are mandatory. However, there is controversy which test batteries and screening methods might be suitable for diagnosing HAND (11–13).

Although the scientific diagnostic criteria for HAND are sometimes not easy to apply in daily practice, PLWH still show or report cognitive impairment. Thus, there is a need for the establishment of a feasible and rapid testing approach to screen PLWH for cognitive deficits and correlate them with possible onset of atrophy, even if the patients are still asymptomatic.

Early brain volume involution in neurodegenerative diseases, as a measure of brain atrophy, can be considered as a potential predictor for cognitive impairment. Moreover, on the basis of the third ventricle or midbrain area (14, 15) differentiation between different forms of dementia are made.

There is evidence that the enlargement of ventricular volume over the time correlates with changes in cognitive function (16). In this regard, transcranial sonography (TCS), which is a non-invasive, side-effect-free method, can be used to examine the third ventricle of the brain (17).

Therefore, we performed a prospective matched-control study to correlate the enlargement of ventricular volume with cognitive function in PLWH. We used TCS to measure the transverse diameter of the third ventricle in unselected PLWH and compared these results with non-infected healthy age- and gender-matched controls. TCS results were correlated with results of neuropsychological testing, laboratory parameters and HIV-specific parameters like CD4-count and CNS penetration-effectiveness (CPE) as a score which depicts the cerebrospinal fluid (CSF) penetration and efficacy of ART in the central nervous system (CNS) (18, 19).

In addition, cranial magnetic resonance imaging (MRI) was used to correlate TCS findings and for the detection of possible brain alteration (global atrophy, strokes, evidence of opportunistic infections). Integrity and brain connectivity were examined by the additional determination of fractional anisotropy (FA) by Diffusion Tensor Imaging (DTI), which has been used before in inflammatory brain diseases like multiple sclerosis (20).

## Patients and methods

### Participants

PLWH were recruited from the outpatient HIV-center of Saarland University Medical School and two private practices. Inclusion criteria were age ≥ 18 years, serological confirmed HIV infection and signed written consent to participate in the study. Pregnancy, cognitive, or emotional inability to sign the informed consent or any concomitant neurological brain disease (e.g., stroke, structural epilepsy, dementia or opportunistic infection) were considered as exclusion criteria. The study was approved by the local ethics committee (Ethikkommission der Ärztekammer des Saarlandes, ethical vote No. 205/17) and conducted in accordance with the Declaration of Helsinki.

### Transcranial ultrasound, magnetic resonance imaging, and Nine-Hole-Peg-Test

Transcranial sonography (TCS) was performed using “MyLab 25 Gold” ultrasound device (Esaote, Germany) with a low-frequency 1-4 MHz transducer PA240. All measurements were performed by two well-experienced examiners (> 10 years of experience). For all measurements, we used a standardized protocol based on previously described parameters and methods (21, 22): sonographic brain imaging started in the axial plane placing the ultrasound probe in the orbitomeatal line. Next, the mesencephalic brain stem was identified and the ultrasound probe was angled upward to display the diencephalic and ventricular plane. The third ventricle appeared as an anechogenic era between two hyperechogenic parallel lines (where the ultrasound beams meet the ependyme in an orthogonal manner). Then, the distance between the inner margins of both lines which form the third ventricle walls was measured in a right angle. Measurements were strictly made in the center of the ventricle. Time of examination was 5–10 min. To eliminate any confounding bias, both examiners were blinded.

To determine the reliability of TCS measurements, MRI was performed using SIEMENS Magnetom Skyra 3T in 72 patients to correlate the results of TCS using standard neuroradiological protocols for measuring the maximal axial diameter of the third ventricle. A T1-MPRAGE (Magnetization Prepared Rapid Acquisition Gradient Echo) and T2-weighted sequences were acquired to detect potential structural changes in the CNS parenchyma, such as signs of atrophy or infarction. Routinely, a T2-FLAIR (Fluid Attenuated Inversion Recovery) and a T2-FLASH (Fast Low-Angle Shot) sequence and a T2-SPACE (Sampling Perfection with Application optimized contrasts using different flip angle evolution) sequence were added. Diffusion-weighted imaging (DWI) and DTI were used to detect diffusion defects and determine fractional anisotropy (FA). FA is considered as measurement of brain integrity, connectivity, and activity of white matter tracts that can be determined by MRI (23). Results were determined from 0 to 1, where a FA value of 1 was equated to maximum anisotropy with complete directionality of diffusion in exactly one direction.

The Nine-Hole-Peg-Test (9HPT), known as a speed measuring pegboard test for the upper extremities in multiple sclerosis patients (24), was used to assess dexterity disorder.

### Neuropsychological testing

Testing for cognitive deficits was carried out using the neuropsychological test battery CERAD-PLUS (Consortium to Establish a Registry for Alzheimer's Disease), which is a tool designed for Alzheimer's dementia assigning deficits to neuropsychological sub-domains such as executive functions and psychomotor speed (21). Domains that are thought to be affected in cognitively impaired HIV patients (9, 25) are also tested with the CERAD plus: Verbal word fluency (semantic word fluency by naming animals within 1 min; phonematic word fluency by S-words), executive functions (for example, by Trail Making Test B), information processing speed (Trail Making Test A), verbal and visual learning (by encoding, retrieving, and memorizing, attention partially by Trail Making test A). All test persons completed the paper-bound test battery with personal instruction. Neuropsychological assessments were performed using Becks Depression Inventory (BDI) for depression screening (26) and the questionnaires Fatigue Severity Scale (FSS) for fatigue (27). Regarding QoL, we used the Short-Form-36 (SF36) (28, 29).

### Statistical analysis

Numerical and descriptive variables are given as means ± standard deviation of the mean. The categorical data are presented as numbers and frequencies. To compare the means of the numerical data, parametric *t*-tests were used. In case of categorical variables, the comparison of proportions was done with chi-squared test. Correlation analysis was performed using the Spearman rank correlation coefficient. For univariate analysis, linear regression was used. All hypotheses were tested at a *p*-value of 0.05. Statistical analysis was performed with SPSS v25.0.0.1 (IBM, Ehningen, Germany).

## Results

In total, 80 people were examined, 52 PLWH (45 males, 7 females) and 28 non-infected healthy controls (23 males, 5 females). Median age of the HIV-cohort was 52 years (24–78 years) and 51 years for the healthy controls (22–79 years), *p* = 0.820. Both cohorts had the same median of comorbidities (1.0) with cardiovascular disease as the most often (refer detailed overview in Table 1). Mean duration of HIV-infection was 11.9 years (range 0–35 years) and mean CD4 lymphocyte count 649/μl (SD ± 271.12). Fifty-one patients (98%) received ART with a mean HIV-1 plasma RNA of 28 copies/ml (range 0–1,100). Evaluation of CD4 lymphocyte nadir was possible in 44 patients with a mean CD4 nadir of 390/μl (SD ± 241.35). In addition, three patients had CD4 lymphocyte count lower than 200/μl. According to the used ART regime, PLWH were divided into three groups: non-nucleoside reverse transcriptase inhibitor (NNRTI) + integrase strand transfer inhibitor (INSTI) (*n* = 41), NNRTI + protease inhibitor (PI) (*n* = 4), and nucleoside reverse transcriptase inhibitor (NRTI) + NNRTI (*n* = 6) (refer Table 1).

**Table 1:** Patients and probands characteristics.

	**PLWH (*n* = 52)**	**Control group (*n* = 28)**	***p***
**Age**^**1**^ years: Median (IQR; Min, Max)	52 (14; 24, 78)	51 (15; 22, 79)	0.820^A^
**Sex**^**2**^: male n (%)	45 (86.5%)	23 (82.1%)	0.599^B^
**Sexual orientation**		
Heterosexual: n (%)	19 (36.5%)	25 (89.3%)	<0.001^B^
MSM homosexual: n (%)	28 (53.8%)	3 (10.7%)	
MSM bisexual: n (%)	5 (9.6%)	0	
**Education**^**3**^ **years:** n (IQR)	13 (4)	12 (3)	0.622^A^
**Patients (n) with pre-existing diseases**	27	1	0.613^A^
Median of pre-existing diseases (IQR)	1 (2)	1 (2)	
Infectious: n (%)	5 (20.0%)	4 (22.2%)	
Cardiovascular: n (%)	12 (48.0%)	6 (33.3%)	
Psychiatric: n (%)	2 (8.0%)	1 (5.6%)	
Neurological: n (%)	3 (12.0%)	3 (16.7%)	
Hemato-oncological: n (%)	7 (28.0%)	2 (11.1%)	
Allergies: n (%)	4 (16.0%)	1 (5.6%)	
Pulmonary: n (%)	2 (8.0%)	1 (5.6%)	
Surgical/trauma: n (%)	3 (12.0%)	2 (11.1%)	
Musculoskeletal: n (%)	7 (28.0%)	2 (11.1%)	
Metabolic: n (%)	1 (4.0%)	7 (38.9%)	
ENT: n (%)	1 (4.0%)	2 (11.1%)	
**CD4 T cell count [/μl]:** mean (Min, Max)	648.6 (812.9, 1299.3)		
**HIV-1 plasma RNA [c/ml]:** mean (Min, Max)	27.5 (0, 1100)		
**Antiretroviral therapy:** ***n*** **(%)**	51 (98%)		
NRTI + NNRTI: n (%)	6 (11.5%)		
NRTI + PI: n (%)	4 (7.7%)		
NRTI + INSTI: n (%)	41 (78.8%)		
**CPE-Score: n (%)**	51 (98%)		
CPE-Score = 7: n (%)	33 (63.5%)		
CPE-Score = 8: n (%)	16 (30.8%)		
CPE-Score > 8: n (%)	2 (3.8%)		

A Mann-Whitney U-Test.

B Chi-Quadrat-Test.

1,2,3 Matching parameters.

### Transcranial ultrasound and neuropsychological testing

**Figure 1:**
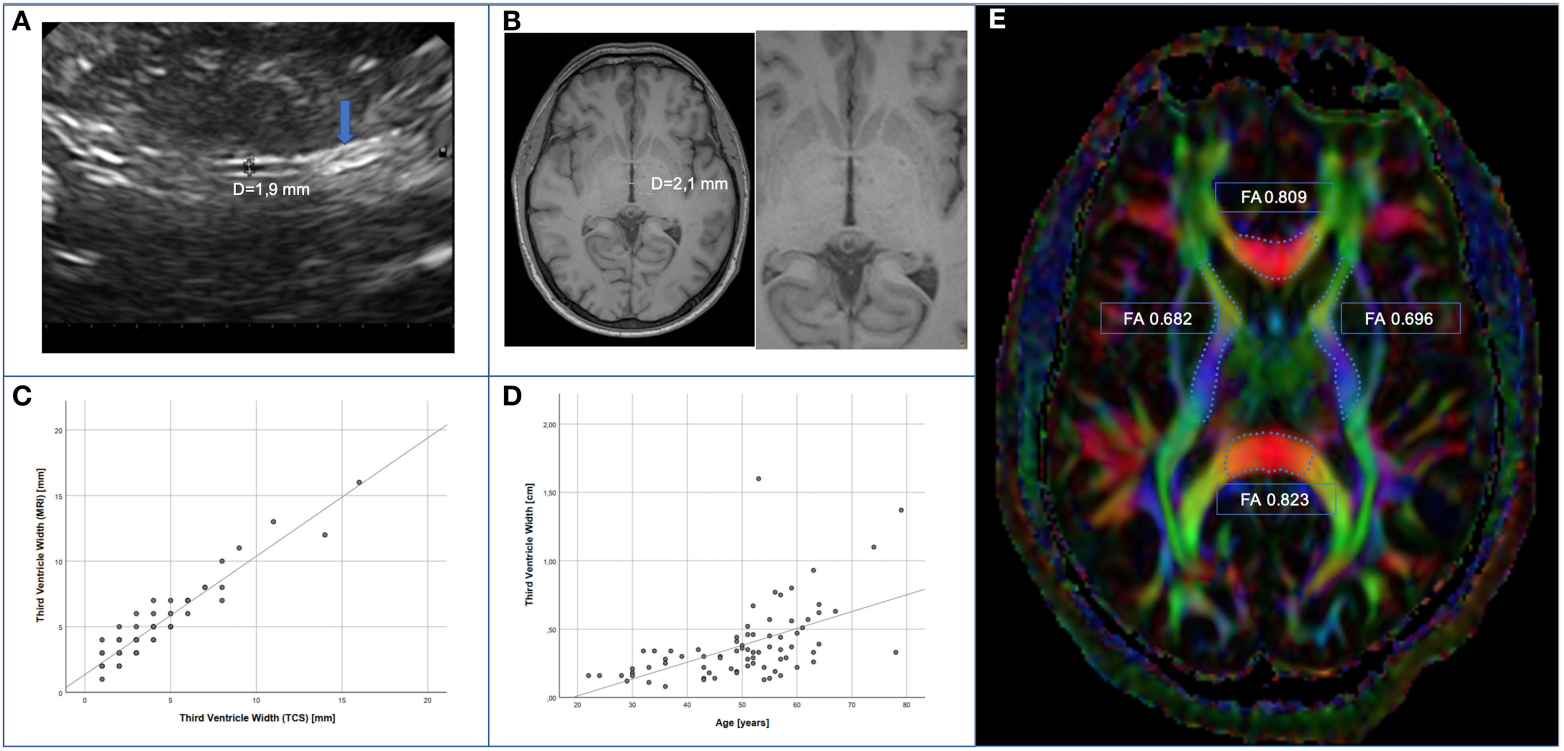
Measurement of WTV. **(A)** Example of sonographic measurement of third ventricle width after identification of the pineal gland (arrow). **(B)** Example of measurement of third ventricle width in axial plane by MRI. **(C)** Correlation of TCS- vs. MRI-based assessment (spearman *r* = 0.884; *p* < 0.001). **(D)** Correlation of TCS based assessment and age (spearman *r* = 0.546; *p* < 0.001). **(E)** illustrative example of FA-measurement of corpus callosum (genu and splenium) and capsulae internae on both sides.

The third ventricle ventricular width (WTV) could be measured by TCS on at least one side in 76 patients (95%). In both cohorts, diameter of the WTV correlated significantly with age (*r* = 0.546; *p* < 0.001) (Figures 1A,D). The analysis showed a trend toward greater ventricular width in the HIV cohort with a median of 3.44 mm (min 0.8 mm; max 16.0 mm) compared with the control cohort with a median 2.8 mm (min 1.1 mm; max 13.7 mm) (*p* = 0.085).

PLWH reported significantly more memory problems (42.3 vs. 10.7%; *p* = 0.004) and poor concentration (30.8 vs. 7.1%; *p* = 0.016) level. A total score was calculated for the CERAD test battery (30). The analysis showed a significant correlation between WTV and the CERAD total score (rho = −0.300; *p* = 0.009), the trail making test A (rho = 0.375; *p* < 0.001) and B (rho = 0.270; *p* = 0.018) results of all patients. Table 2 illustrates the results of the CERAD plus test battery. In the HIV-cohort, there was a trend to a lower CERAD total score and better trail making test B, without reaching statistical significance in the total group comparison (*p* = 0.280) (Table 2). The control group showed significantly better results in the trail making test A (*p* = 0.030) and the phonematic word fluency results (*p* = 0.024). Comparing only 25 matches (same age and gender and same education level each of 25 PLWH and 25 controls), there was also no statistically significant difference in total score (*p* = 0.142). Again, the control group performed slightly better in the subgroup analysis in verbal fluency (*p* = 0.015) and trail making test A (*p* = 0.047; d = 0.285) items, and in the mini-mental status test (*p* = 0.044). To check the integrity of the raw data, so-called Z values were determined by comparison with normalized data sets. However, this was only possible for persons aged 49 and older. In the group comparison of these Z-scores, there were no statistically significant differences for items of the CERAD Plus battery.

**Table 2:** Comparison of the results (raw scores) of PLWH and control group in the CERAD plus test battery using Mann-Whitney U-test.

	**PLWH (*n* = 52)**	**Control group (*n* = 28)**		
	**Median [Min; Max]**	**Median [Min; Max]**	***p***	**Cohens d**
CERAD total score	93.50 [67; 109]	97.50 [79; 107]	0.28	
Verbal fluency	21.00 [8; 34]	24.00 [12; 32]	0.024	0.252
Boston naming test	15.00 [13; 15]	15.00 [13; 15]	0.065	
Mini-mental state examination	28.50 [26; 30]	29.00 [24; 30]	0.094	
Wordlist learning	22.00 [14; 28]	22.00 [14; 27]	0.968	
Wordlist Recall	8.00 [4; 10]	8.00 [3; 10]	0.996	
Wordlist recognition	20.00 [16; 20]	20.00 [18; 20]	0.947	
Constructional praxis	11.00 [6; 11]	11.00 [9; 11]	0.121	
Constructional praxis recall	11.00 [5; 11]	11.00 [2; 11]	0.658	
Trail Making Test A	41.00 [19; 86]	33.00 [17; 108]	0.030	0.243
Trail Making Test B	77.50 [22; 258]	65.50 [28; 300]	0.073	
S-Words (phonematic fluency)	11.00 [5; 22]	12.00 [4; 21]	0.131	

The CERAD total score includes all items except S-words and the Trail Making tests.

In order to detect dexterity disorder as it may occur in HAND, the 9HPT was added, which showed no differences in both groups.

### MRI parameters, MRI-based measurement of third ventricular width and fractional anisotropy

The proportion of MRIs without any abnormalities (such as atrophy, leukoencephalopathy, or expired strokes) was the same (PLWH 47.1%, controls 48.1%), additionally similar levels of cerebral microangiopathy (PLWH 27.5%, controls 33.3%) and age-related global brain volume involution (PLWH 19.6%, controls 22.2%) were found in both groups. In order to avoid alleged accuracy, MRI-assessed values of WTV were adjusted upward. For method comparison, TCS-based values were adjusted upward, too. As the values were not normally distributed, Spearman rank correlation was calculated. We found a statistically significant correlation between MRI- and TCS-based assessment of WTV in 72 patients (*r* = 0.884; *p* < 0.001) (Figures 1B,C).

Fractional anisotropy as a marker of cerebral parenchymal integrity was examined by MRI in the DTI (Figure 1E). Corpus callosum (genu and splenium) and capsulae internae on both sides were measured. The FA values – given as median [min; max] – for genu corporis callosi (PLWH: 0.712 [0.590; 0.809]; controls: 0.718 [0.497; 0.800]), splenium corporis callosi (PLWH: 0.785 [0.633; 0.895], controls 0.797 [0.652; 0.861]), capsula interna right (PLWH: 0.666 [0.583; 0.730]; controls: 0.666 [0.625; 0.761]), and capsula interna left (PLWH: 0.668 [0.579; 0.778]; controls: 0.662 [0.573; 0.716]) did not differ significantly between the two groups (*p* > 0.05). Only in the genu corporis callosi of all subjects, a statistically significant negative correlation of FA with age was found (rho = −0.235; *p* = 0.042). Within the HIV-cohort, the duration of HIV disease had no significant influence on FA, but the regression analysis showed a significant influence of the CD4 nadir (*R* = 0.345, *p* = 0.025) on FA of the splenium corporis callosum and a non-significant trend on FA of the genu corporis callosum (*R* = 0.299, *p* = 0.055).

This is consistent with the observation in our HIV cohort that poor test scores in the CERAD total score (and in some subsets of the CERAD-PLUS test battery) correlate with decreased FA scores in the genu corporis callosi (rho = 0.405, *p* = 0.004). Negative correlations were found between trail making test A and FA of the genu corporis callosi (rho = −0.281, *p* = 0.048) and FA of splenium corporis callosi (rho = −0.341, *p* = 0.015) as well as trail making test B and FA of the splenium corporis callosi (rho = −0.316, *p* = 0.025).

### Influence of HIV-specific variables on WTV

The duration of HIV-disease had no influence on WTV in the regression analysis (*p* = 0.224). Only three patients had a current CD4 lymphocyte count lower than <200/μl. These three patients had a mean third WTV of 3.3 mm (SD ± 1.57). Patients with CD4-count > 200/μl had a mean third ventricle width of 4.1 mm (SD ± 2.83), but were older of age (mean age 50.8 vs. 42.3 years). Linear regression analysis showed no influence of compromised immune system defined as low CD4-Count <200/μl (*p* = 0.632) on WTV.

**Figure 2:**
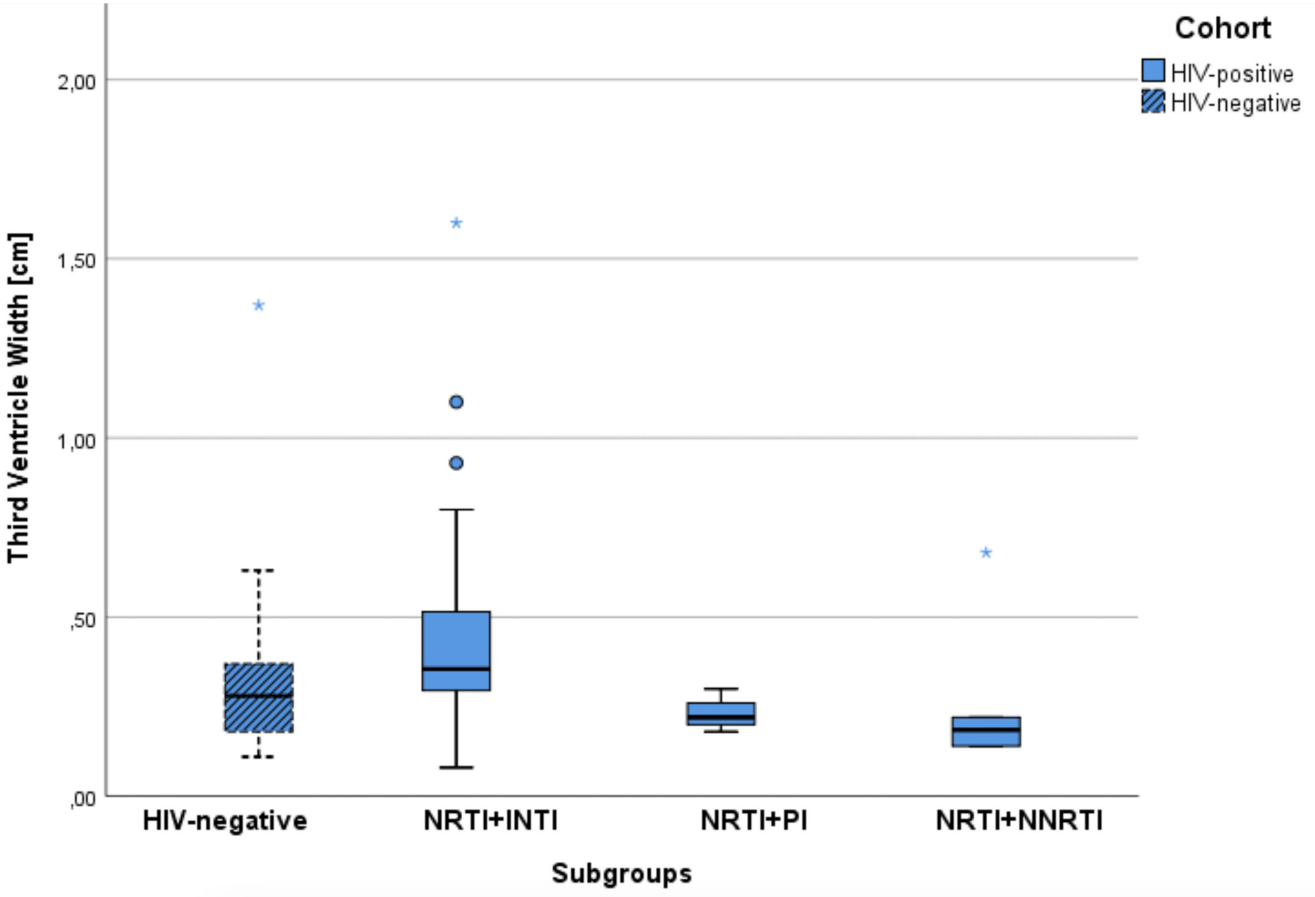
Comparison of median width of the third ventricle for the control group and the ART subgroups of PLWH.

CD4-nadir <200/μl in 9 patients with mean age of 41.1 ± 12.15 years had no influence on WTV (mean WTV of 3.0 mm, SD ± 1.21; *p* = 0.228). Patients with CD4-nadir > 200/μl had a mean WTV of 4.4 mm (SD ± 2.87), but were significantly older of age (mean age 52.8 ± 8.64). Similarly, no significant correlation was found between CD4 nadir and CERAD items. The CSF penetrability of ART, measured by the CPE score (CPE score = 7 and CPE score >7), correlated neither with the CERAD total score (*p* = 0.801) nor with WTV (*p* = 0.424). Regarding ART group comparison NRTI-NNRTI (mean age 50.0 years) vs. NRTI-INSTI (mean age 52.2 years): the NRTI-NNRTI group showed a statistically significant lower WTV of 1.85 mm vs. 3.55 mm (*p* = 0.024) (Figure 2).

### Neuropsychological test results: Quality of life, depression, and fatigue

PLWH performed statistically significantly worse in the items “Social functioning” (*p* = 0.019), “Psychological wellbeing/Mental health” (*p* = 0.001), “Vitality” (*p* = 0.028), and “General health perception” (*p* = 0.018) of the SF36 reflecting QoL (Table 3).

**Table 3:** Results of the SF-36 related to the dimensions in the group comparison in the Mann-Whitney U-test.

	**PLWH (*****n*** **=** **52)**		**Control group (*****n*** **=** **28)**		
	**Median**	**[Min; Max]**	**Median**	**[Min; Max]**	***p***	**Cohens d**
Physical functioning	88.88	[11.11; 100.00]	94.44	[27.77; 100.00]	0.075	
Role limitation (physical)	100.00	[0; 100.00]	100.00	[0; 100.00]	0.170	
Physical pain	87.50	[12.50; 100.00]	90.00	[0; 100.00]	0.871	
Social functioning	87.50	[0; 100.00]	100.00	[50.00; 100.00]	0.019	0.263
Mental health	74.00	[24.00; 100.00]	88.00	[48.00; 100.00]	0.001	0.383
Role limitation (emotional)	100.00	[0; 100.00]	100.00	[0; 100.00]	0.051	
Vitality	60.00	[5.00; 95.00]	67.50	[10.00; 100.00]	0.028	0.246
General health	65.00	[10.00; 100.00]	75.00	[53.00; 100.00]	0.018	0.265
Health change	50.00	[0; 100.00]	50.00	[25.00; 100.00]	0.511	

Higher scores reflect a better quality of life.

**Figure 3:**
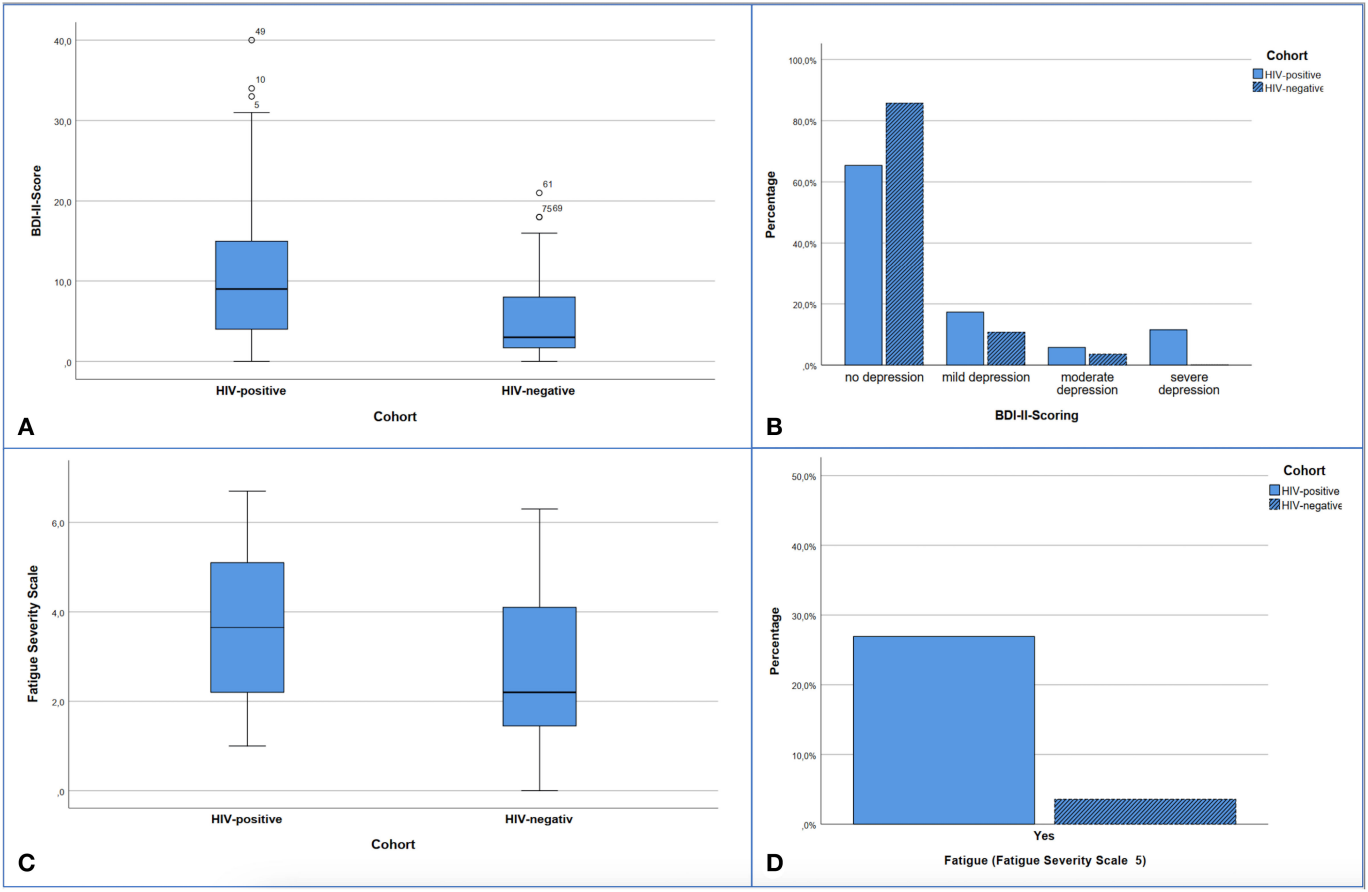
Presentation of screening results for depression **(A,B)** and fatigue **(C,D)**. **(A)** Significant difference with *p* < 0.005 in median comparison of BDI-II Scores: HIV-positive 9.0 (Min 0; Max 40.0) vs. HIV-negative 3.0 (Min 0; Max 21.0). **(B)** Distribution of depression levels according to the BDI-II within the cohorts. 14.3% of the control subjects and 34.6% of the HIV subjects showed depressive symptoms (cut-off 13.0 points). 0-−12 no depression or clinically unremarkable, 13–19 mild depressive syndrome, 20–28 moderate depressive syndrome, >29 severe depressive syndrome. Significant difference in Mann-Whitney-U-test with *p* = 0.040. **(C)** FSS points comparing both cohorts, significant difference with *p* = 0.013 in median comparison: HIV-positive: 3.65 (Min 1.0; Max 6.7) vs. HIV-negative 2.20 (Min 0; Max 6.3). **(D)** Proportion of subjects within the cohorts with fatigue detected in the FSS (cut-off > 5), significant difference with *p* = 0.028 by Chi-square test.

Furthermore, PLWH had a statistically significantly higher BDI score (*p* = 0.005) and FSS score (*p* = 0.013) (Figure 3). These both symptom scores correlated with each other in the HIV-cohort (rho = 0.693; *p* < 0.001) as well as control group (rho = 0.467; *p* = 0.012). However, in both cohorts, the level of depression (HIV cohort *p* = 0.906, control *p* = 0.897) and the FSS score (HIV cohort *p* = 0.203, control *p* = 0.583) had no effect on the CERAD Total Score.

## Discussion

Since the beginning of the AIDS pandemic cognitive deficits have been an issue for PLWH (31) even though, e.g., HAD, which is the most severe form of HAND, was identified before the modern ART era. As ART improved over the last decade with now higher CNS penetration level, it is crucial to use accurate neurocognitive testing and brain imaging when there is evidence of neurocognitive deficits, neuropsychological abnormalities, and fatigue. Therefore, we examined PLWH who were treated with modern ART regarding above-mentioned deficits. Previous studies revealed WTV as a predictor of cognitive deficits in degenerative neurologic diseases (16). Therefore, we aimed to prospectively asses WTV in PLWH compared to a non-infected (age and gender matched) control group. In brief, PLWH reported significant more memory deficits (*p* = 0.004) accompanied by a poor concentration (*p* = 0.016) level. However, there was only a trend for a low score in the CERAD total score in the PLWH group, and the control group performed significantly better in only two subgroups of the test battery.

Although CERAD originally examined cognitive deficits commonly seen in Alzheimer's disease, such as memory impairment, disorientation, loss of expressive and receptive language, and dyspraxia (21), by adding the PLUS subtests the domains can be expanded so that the CERAD Plus batterie also appears suitable for assessing cognitive deficits of other causes. The CERAD Plus has been used for cognitive assessments in other degenerative diseases like Parkinson's disease (32, 33), inflammatory diseases like Neuro-Sjögren's syndrome (34), in patients with microbleeds after stroke (35), or middle-aged adults with metabolic syndrome with total score (36) underlining its broad applicability.

The use of the CERAD plus test battery also proved to be very useful and time-saving for our analysis and covered most domains (required for the diagnosis of HAND) (9); hence, working memory alone was not well-represented. In particular, the use of a CERAD total score, as also mentioned in previous publications (37), provides a good initial overview of global cognitive outcomes, although other test batteries are often used in large cohort studies (such as Multicenter AIDS Cohort Study) (29, 38).

Alternative scales such as the HIV dementia scale, Western Neuropsychological Test Battery (39), or the international HIV dementia scale (40), which were used to identify HAD patients, might need to be reassessed during the period of adequate ART therapy (12) or the establishment of new cut-off values (41).

Recent studies have seen similar results regarding memory deficits in PLWH (41), although we did not use the recent published Frascati-criteria – which use yielded a highly variable prevalence in a meta-analysis for the diagnosis of HAND (42) – which would have included a detailed history of limitations in the instrumental activities of daily living (IADL) (9, 43). We did not systematically investigate these IADLs; on the other hand, the initially taken medical history did not reveal any relevant deficits in the areas that made the presence of MND or HAD a priori unlikely.

In our study, CD4 T-cell count was not a significant determinant of neurocognition. Nevertheless, other recent studies showed that both higher CD4 T-cell count and longer ART duration were associated with lower neurocognitive impairment, measured by the Modified Mini Mental State (M3S) (44). Deficits in similarity assessment were most common. To evaluate whether CNS penetration level of the given ART regimes had an impact on subtle neurologic disorders in our analyzed patient cohort, the taken ART regimens were analyzed regarding their CNS Penetration-Effectiveness (CPE) score (45). As all ART regimes had a CPE score > 7, there was no statistically significant worse outcome in overall neurocognitive performance in our HIV cohort compared to the control group may reflecting the good CNS penetration of the taken ART regimens. This is of interest, as earlier studies have shown that poorer CNS penetration of ART appears to allow continuing HIV replication in the CNS (45) which might contribute to neurologic disorders in PLWH. Therefore, it is conceivable that the reported cognitive impairment in our cohort might be associated with depression or fatigue. 9HPT could not detect dexterity disorder as a possible symptom in HAND, although the early slowing of rapid movements (of the previously termed HIV dementia complex) should no longer be expected as an early symptom under sufficient ART as it was only described in older publications (46, 47).

The WTV of all study participants correlated significantly with overall neurocognitive performance (CERAD total score and trail making tests). Our results (regarding WTV in PLWH) are in line with previous imaging studies investigating possible correlations between markers of brain atrophy and cognitive deficits in patients with degenerative brain disease (such as Parkinson's disease) (14, 48) and also HIV patients: Yaldizli et al. found in 2006 in their HIV cohort with a comparable mean age of 46 ± 12 years a similar mean diameter of the third ventricle of 4.4 ± 2.5 mm comparable to the one in our own cohort (49). In contrast to Yaldizli, now 12 years later, we only measured a trend toward WTV enlargement in PLWH without correlation to HIV-specific characteristics. This may reflect the improved efficacy of ART including high CNS penetration shown by a high CPE-score (45) in our cohort. In line, studies of cohorts in developed countries suggest that ART with better CNS penetration might be associated with reversal or even prevention of neurocognitive dysfunction (50), whereas there is evidence of increased prevalence of HAND in sub-Saharan Africa, for example (51).

Patients with HIV and low CD4-counts as far as advanced CDC stages are more likely to develop HAND (52). Nevertheless, the duration of HIV-infection had no impact on WTV in our cohort. Moreover, we found no correlation between CD4-count and WTV. HIV-1 RNA was <50 copies/ml in 48 (92%) patients reflecting successful viral suppression which may explain that we found no correlation between HIV plasma viral load and WTV.

Regarding the TCS, recent studies showed that TCS is an easily accessible tool to measure WTV (53). We found a high interrater reliability and correlation between TCS and MRI measurements of WTV confirming previous findings in PLWH (49) as far as in neuroinflammatory disease like multiple sclerosis (54). Future studies must show if WTV really has the potential to play a significant role in the diagnosis of neurocognitive dysfunction or HAND in PLWH. Because of its simplicity, it might be used as an easy regular monitoring even before objectifiable cognitive deficits appear. If it turns out that WTV does not deviate significantly from healthy control subjects, it should be discussed whether this is caused by modern ART. Moreover, it is questionable whether sonographic measurement of the ventricle is sensitive enough and expedient at all. In contrast, MRI has the advantage that it also examines the brain parenchyma. Decreased FA was found to result in poorer global cognitive performance, processing speed, and executive functions, performance (55). These affected domains as well as impaired attention and working memory were often reported in HIV patients in the pre-ART era (56). However, the extent to which fractional anisotropy can be used as a possible parameter for sub-parameters of neurocognitive performance or fatigue or affective disorders must be shown in prospective studies with larger numbers of study participants; although there is evidence that decreased fractional white matter anisotropy may be associated with cognitive deficits in PLWH (57).

In our study, there were no differences in FA in both groups. Hence, Davies et al. found decreased FA in the internal capsule, right corpus callosum, and other regions, but in their cohort cognitive impairment was 28 vs. 5% in controls (58). Another study by Towgood et al. found no significant differences in neurocognitive testing between HIV-negative and HIV-positive individuals on sufficient ART. Decreased frontal brain volume and reduced anisotropy were found here in the subgroup comparison in HIV-positive older participants (50–75 vs. 20–40 years) (59).

In the future, MRI enhancements with fixation-based analysis (FBA) and free water corrected DTI (60) or a changing pattern in glymphatic clearance function detected by DTI (61) may also be used to improve scientific understanding of cognitive deficits in PLWH.

The PLWH in our cohort had more depressive symptoms shown by a statistically significantly higher BDI-II score (*p* = 0.005) and in addition were suffering more from fatigue as indicated by a higher FSS score (*p* = 0.013). These findings are in line with previous data as there are several studies reporting that depression is two to three times more common in PLWH in comparison with the general population (62, 63) and fatigue is one of the most frequently HIV-associated reported symptom in PLWH (64, 65). Additionally, depression seems to be one of the most common neuropsychiatric disorders in PLWH (64). While one study group found that higher levels of depression predict more significant deficits in global cognitive functioning (especially motor functioning and processing speed) (66), no significant correlation was found in our study. Hence, it has to be kept in mind that the above-mentioned cohort studied by Bryant et al. included patients with histories of alcohol and cocaine abuse which might contribute to deficits in global cognitive function itself (66). Regarding QoL, PLWH performed statistically significantly worse in the items “Social functioning” (*p* = 0.019), “Psychological wellbeing” (*p* = 0.001), “Vitality” (*p* = 0.028), and “General health perception” (*p* = 0.018) of the SF36. However, evidence of stigma or discrimination, which had recently been identified as predictors of impairment in health-related QoL (67) was not found in our cohort.

Hence, our study does have some limitations. First, its monocentric design and the small number of included patients probably underestimate the effect of WTV as a marker for mild or asymptomatic neurocognitive disorders in PLWH. Second, due to COVID-19 pandemic, we were only able to include 28 people in the control group. However, our findings are clinically valuable because it is the first study on the use of TCS in the modern ART era as a screening tool for WTV in PLWH. Therefore, our study warrants further analysis regarding TCS as a screening tool for neurocognitive disorders in PLWH and should be therefore validated in future longitudinal studies.

All together, we have shown that PLWH, who have regular access to sufficient ART with high CPE score, are less likely to have objectifiable cognitive deficits as measured by neuropsychological tests – as compared to previous studies of PLWH – and less brain atrophy as measured by third ventricle sonography and MRI. This may indicate no increased risk of dementia compared to the healthy population. Still, limitations in their daily life often result from comorbid depression and fatigue. We suggest that TCS as a fast-performed and easily accessible screening tool for neurocognitive deficits in PLWH should be validated in future longitudinal studies.

## Data availability statement

The raw data supporting the conclusions of this article will be made available by the authors, without undue reservation.

## Ethics statement

The studies involving human participants were reviewed and approved by Ethikkommission der Ärztekammer des Saarlandes, ethical vote No. 205/17. The patients/participants provided their written informed consent to participate in this study.

## Author contributions

DK-M, MH, MB, UY, KF, SB, and MF contributed to collection, review, and analysis of the data. GW and MH performed statistical analysis. DK-M and MF wrote the manuscript. All authors contributed to the article and approved the submitted version.

## Conflict of interest

The authors declare that the research was conducted in the absence of any commercial or financial relationships that could be construed as a potential conflict of interest.

## Publisher's note

All claims expressed in this article are solely those of the authors and do not necessarily represent those of their affiliated organizations, or those of the publisher, the editors and the reviewers. Any product that may be evaluated in this article, or claim that may be made by its manufacturer, is not guaranteed or endorsed by the publisher.
